# Minimal residual disease assessment through ctDNA facilitates tailored immunotherapy in MSI-high, NTRK1-fusion pancreatic adenocarcinoma

**DOI:** 10.1093/oncolo/oyag108

**Published:** 2026-03-30

**Authors:** Sarah Rohlfing, Dilyana Vladimirova, Stephanie Berger, Sylvia Bochum, Gabrijela Otmacic, Marlene Weiß, Bence Sipos, Saskia Biskup, Marion Klaumünzer, Uwe M Martens

**Affiliations:** Clinic for Gastroenterology, Hemato-Oncology, Pneumonology, Infectiology, and Intensive Care Medicine, RKH Fürst-Stirum-Klinik Bruchsal, 76646, Germany; Department for Hematology, Oncology, and Palliative Medicine, SLK-Clinics Heilbronn GmbH, 74078, Germany; Department for Hematology, Oncology, and Palliative Medicine, SLK-Clinics Heilbronn GmbH, 74078, Germany; Department for Hematology, Oncology, and Palliative Medicine, SLK-Clinics Heilbronn GmbH, 74078, Germany; Department for Hematology, Oncology, and Palliative Medicine, SLK-Clinics Heilbronn GmbH, 74078, Germany; Department for Hematology, Oncology, and Palliative Medicine, SLK-Clinics Heilbronn GmbH, 74078, Germany; Qualipath—Practice for Pathology and Molecular Pathology, Stuttgart, 70176, Germany; Center for Human Genetics Tübingen, Tübingen, 72076, Germany; Center for Human Genetics Tübingen, Tübingen, 72076, Germany; Department for Hematology, Oncology, and Palliative Medicine, SLK-Clinics Heilbronn GmbH, 74078, Germany; MOLIT Institute for Personalized Medicine, Heilbronn, 74076, Germany

**Keywords:** PDAC, NTRK fusion, liquid biopsy, minimal residual disease, tailored treatment

## Abstract

Pancreatic cancer remains one of the most lethal malignancies, with limited integration of precision oncology into routine clinical care. We present a unique case of a RAS wild-type, MSI-H, TMB-H pancreatic ductal adenocarcinoma harboring a *TPM3-NTRK1* fusion, monitored through 13 serial liquid biopsies over 3 years. Dynamic changes in *NTRK1*-fusion allele frequency, tumor mutational burden, and the emergence of an *NTRK1* resistance mutation guided finely tuned, situation-adapted therapeutic adjustments: rapid disease control with targeted NTRK inhibition followed by durable remission under immune checkpoint blockade. This case highlights the power of comprehensive molecular profiling and high-frequency ctDNA monitoring to capture tumor evolution and minimal residual disease. Importantly, it further demonstrates how MRD-guided surveillance enables a precise balance between fast-acting targeted therapy and the sustained effects of immunotherapy, providing a blueprint for individualized, context-driven treatment strategies in rare molecular subtypes of pancreatic cancer.

Key Points

- This case illustrates a rare combination of molecular alterations leading to sequential clinical decision-making in the presence of multiple therapeutic targets.
- Longitudinal monitoring via liquid biopsy enabled an adaptive treatment strategy guided by molecular response and remission.
- Recurring molecular tumor board presentations served as a central control instrument throughout the disease course.

## Introduction

Pancreatic ductal adenocarcinoma (PDAC) accounts for >90% of pancreatic malignancies and remains a lethal solid tumor. Chemotherapy can delay progression, but its impact on long-term outcomes remains modest, with a 5-year overall survival <5%.^1^ Thus, molecular testing is employed to identify rare, targetable alterations. Although the majority of PDACs harbor non-targetable *KRAS* mutations, a small subset (5%-10%) arises on the basis of potentially targetable alterations, including gene fusions, mismatch repair deficiency, and alterations in DNA damage repair pathways.^2^ The detection of alterations can enable tailored treatment; however, once a target is identified, it becomes important to monitor its course under therapy. In this context the concept of minimal residual disease (MRD), long established in hematological malignancies, is gaining attention in solid tumors. Emerging data suggest that serial liquid biopsy (LB) enables earlier molecular detection, supporting treatment assessment beyond imaging and timely adjustment to molecular changes.^3^ Here, we describe a PDAC patient for whom molecular analysis directly influenced treatment decisions over 3 years. The case illustrates how integrating molecular diagnostics may help tailor treatment when conventional therapy is unlikely to achieve benefit.

## Patient story

A 53-year-old man with no family history of cancer was diagnosed with advanced PDAC (T4N2M0) after presenting with 20 kg weight loss in August 2021. Baseline imaging revealed a 4.7 × 4.8 cm mass in the pancreatic head abutting the superior mesenteric artery, as well as multiple enlarged peripancreatic lymph nodes. Endoscopic biopsy confirmed adenocarcinoma. Immunohistochemistry (IHC) showed positivity for CK7 and CDX2, while CK20, synaptophysin, and chromogranin A were negative. Initial molecular analysis revealed a *RAS* wild-type (WT) as well as a microsatellite instability (MSI-H). The tumor was considered inoperable. The patient started FOLFIRINOX in August 2021 and switched to gemcitabine and nab-paclitaxel in November 2021 due to progression. Chemotherapies were poorly tolerated, resulting in a 10 kg weight loss, fatigue, and abdominal pain.

## Molecular tumor board

**Figure 1.**
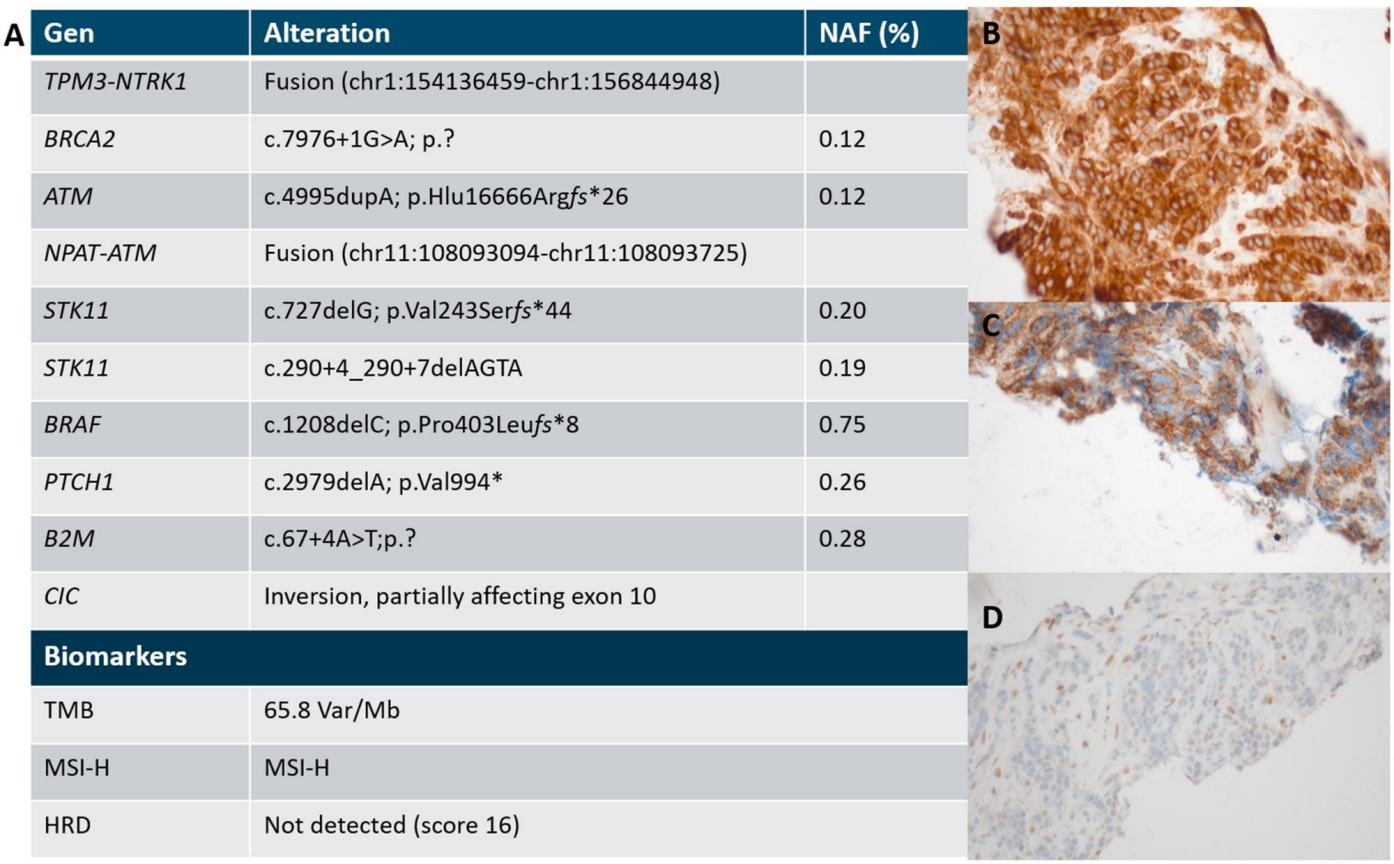
Molecular characterization after progression on second-line standard therapy guiding personalized treatment recommendations. (A) Comprehensive genomic profiling using a 794-gene panel identified a *TPM3–NTRK1* fusion as the principal actionable alteration. Additional somatic variants were detected in *BRCA2, ATM, STK11, PTCH1, BRAF, B2M* and *CIC*. The tumor demonstrated microsatellite instability (MSI-H) and a high tumor mutational burden (TMB). Germline testing identified no (likely) pathogenic mutation or variant of unknown significance. (B–D) Immunohistochemistry (IHC) provided orthogonal validation of key NGS findings: (B) diffuse NTRK protein expression confirming the presence of an oncogenic NTRK fusion; (C) partial retention of β2-microglobulin expression; and (D) loss of ATM protein consistent with the detected ATM alteration. HRD, homologous recombination deficiency; NAF, novel allele frequency; Var/Mb, variants per megabase.

Following the recommendation of the molecular tumor board (MTB), the patient underwent a pancreatic biopsy for NGS analysis in March 2022. The sequencing results revealed a *RAS* WT tumor with a *TPM3–NTRK1* gene fusion (*TPM3* exons 1-8 NM_001364680.2; *NTRK1* exons 12-17; NM_002529.4). Further somatic alterations were detected in *BRCA2, ATM, STK11, PTCH1, BRAF, B2M,* and *CIC* (Figure 1). MSI-H was confirmed, resulting in a high TMB with 65.8 Var/Mb. No pathogenic germline mutation was found. Orthogonal IHC validation confirmed the expression of NTRK protein and demonstrated a functional loss of ATM and retained β2-microglobulin (β2M) expression (Figure 1).

In May 2022, a CT showed progression on second-line chemotherapy. Given the different targeted therapeutic options, the case was re-discussed by the MTB. Following consensus recommendation, the patient was enrolled in a tumor-agnostic clinical trial (TAPISTRY NCT04589845) and started treatment with the NTRK inhibitor entrectinib.

To monitor response and evaluate resistance, serial LBs were collected and analyzed using the Guardant360 CDx assay, enabling assessment of circulating tumor DNA (ctDNA) derived from cell-free DNA (cfDNA). A baseline LB prior to entrectinib confirmed the *NTRK1* fusion and MSI-H status, with a blood tumor mutation burden (bTMB) of 88.9 Var/Mb. MSI-H and bTMB matched those of the tissue analysis, but the mutation profile differed, with only the *TPM3–NTRK1* fusion and the *STK11* mutation shared (Figure 3). Additionally, the patient’s baseline was assessed using the EORTC QLQ-C30 questionnaire, revealing a reduced global health status score of 40.

## Patient update

**Figure 2.**
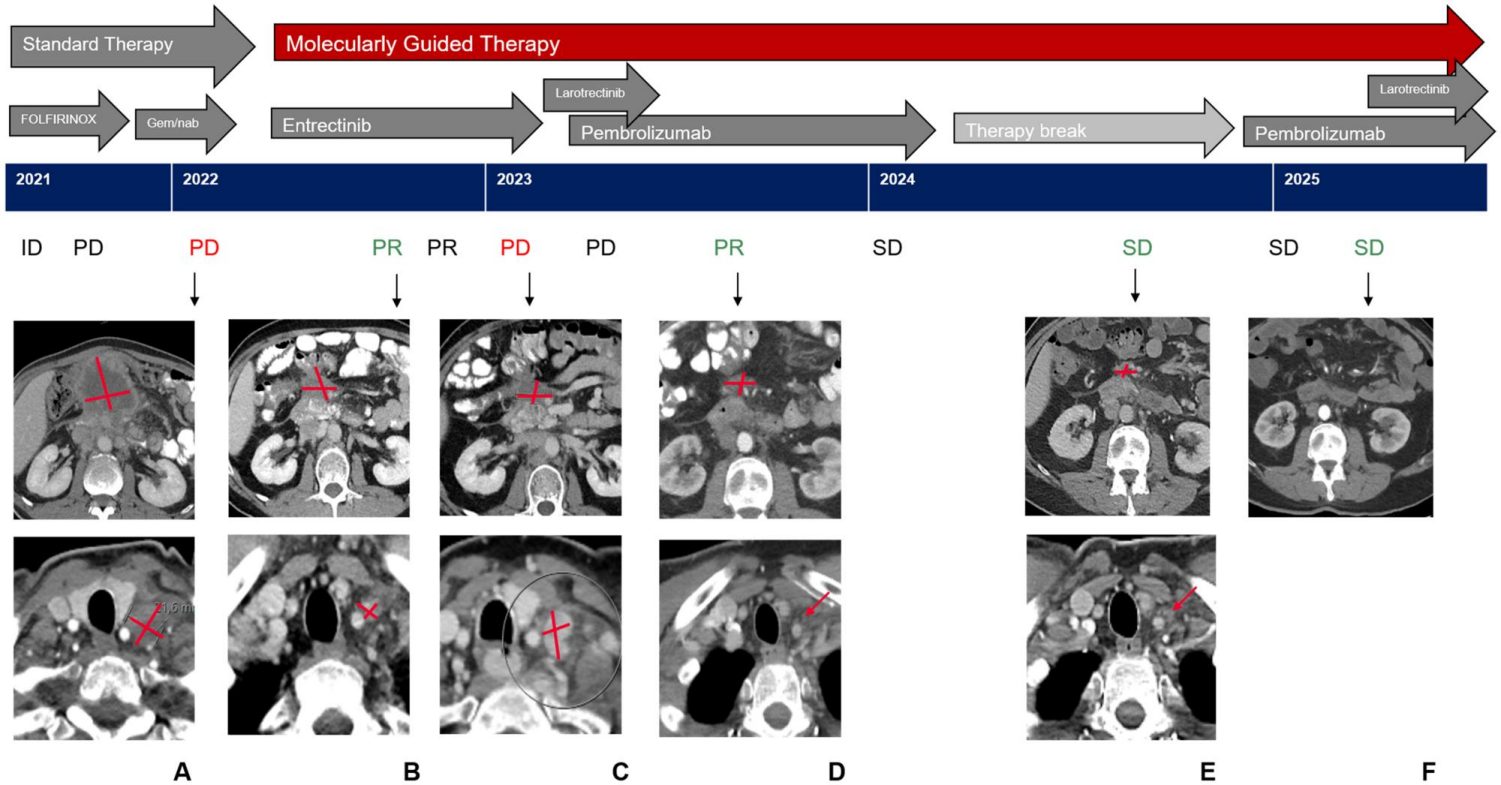
Clinical course under targeted therapy, immunotherapy, and combination treatment. (A): Baseline CT imaging of the abdomen/pelvis and neck prior to the initiation of targeted therapy showed a pancreatic head mass with associated pathological cervical lymphadenopathy. (B): After 16 weeks of entrectinib, CT imaging demonstrated a marked reduction in the size of the primary pancreatic lesion as well as regression of cervical lymph node metastases. (C): At 32 weeks after start of entrectinib CT scans revealed an increase in the size of left cervical lymph nodes, (enlarged supraclavicular, mediastinal and retroperitoneal lymph nodes are not shown). CT scans of abdomen/pelvis and neck 16 weeks after start of pembrolizumab (D), during a therapy break (E) and after resumption of pembrolizumab/larotrectinib show near complete response to therapy. Gem/nab, gemcitabine/nab-paclitaxel; ID, initial diagnosis; PR, partial response; PD, progressive disease; SD, stable disease.

**Figure 3.**
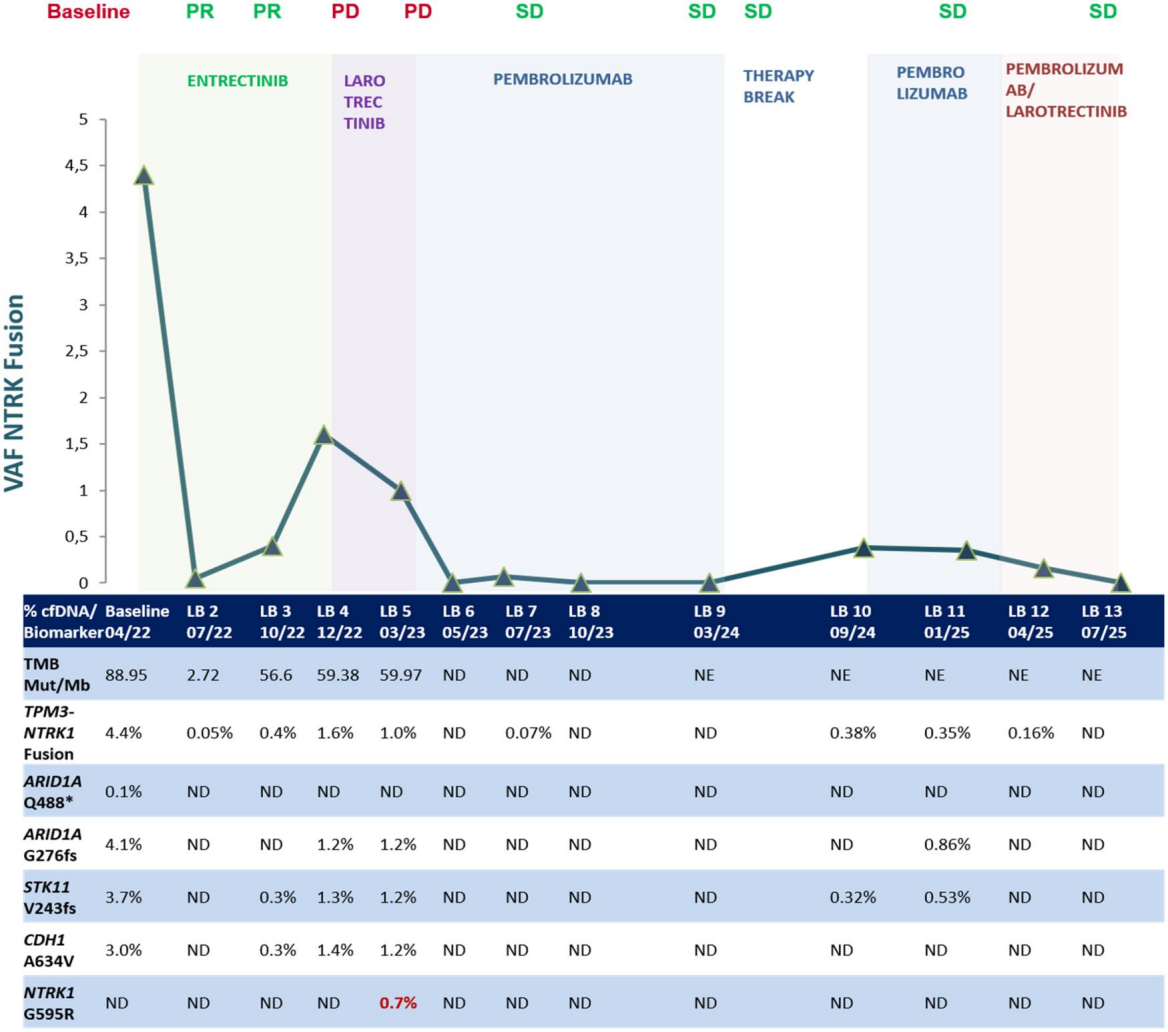
Longitudinal liquid biopsy monitoring to assess treatment response and guide personalized therapy. Baseline cfDNA profiling detected a *TPM3–NTRK1* fusion (VAF 4.4%), microsatellite instability-high (MSI-H), high tumor mutational burden (TMB-H), and mutations in *ARID1A, STK11*, and *CDH1*. Following initiation of TRK inhibition, a rapid decline in *NTRK1* VAF and TMB was observed, consistent with early clinical benefit. After approximately 5 months of entrectinib (Test 3), rising tumor-associated cfDNA levels and increasing TMB indicated emerging molecular progression despite stable CT findings. At 10 months (Test 5), an on-target resistance mutation (*NTRK1* G595R) was detected, prompting discontinuation of TRK inhibition. Pembrolizumab therapy subsequently induced a deep response, reflected by radiologic improvement and complete clearance of tumor-associated cfDNA (Tests 6–9). During a temporary treatment pause, tumor-associated cfDNA levels rose again (Test 10), suggesting molecular relapse before overt radiologic progression. To prevent clinical recurrence, pembrolizumab was resumed and combined with larotrectinib. As of July 2025 (Test 13), cfDNA analysis demonstrated complete molecular remission with undetectable levels of all tumor-derived alterations. LB, liquid biopsy; ND, not detectable; NE, not evaluated; PR, partial response; PD, progressive disease; SD, stable disease; VAF, variant allele frequency.

Imaging after 8 weeks of entrectinib revealed rapid response with a decrease in pancreatic mass and enlarged lymph nodes (Figure 2). The contemporaneous LB demonstrated a decrease in bTMB and in the variant allele frequency of the *TPM3-NTRK1* fusion in cfDNA (from 4.4% to 0.05%) compared to baseline (Figure 3). The other baseline mutations and MSI-H were no longer detectable. Clinical response was rapid with symptom resolution within 14 days. The patient presented in excellent condition, pain-free, with a 5 kg weight gain and markedly reduced abdominal tumor palpability.

Serial CT scans demonstrated a very good partial response as the best response until December 2022. At that time, after 32 weeks of entrectinib, imaging revealed a progressive disease characterized by pathological lymphadenopathy in the cervical, mediastinal, and retroperitoneal regions (Figure 2). However, a significant increase in ctDNA levels and TMB had already been detected in October 2022, 24 weeks after the initiation of NTRK inhibition. Notably, molecular progression occurred about 8 weeks before radiological progression (Figure 3).

The case was repeatedly discussed in the MTB at key decision points. As resistance emerged clinically, the patient was withdrawn from the trial, and entrectinib was switched to another NTRK inhibitor, larotrectinib. At this point, ctDNA levels had increased markedly and remained at high levels after larotrectinib administration. Moreover, shortly after the switch to larotrectinib, an acquired resistance mutation in *NTRK1*, p. G595R, arose in the LB, resulting in the decision to discontinue NTRK inhibition. In addition, a significant increase in ctDNA levels, TMB, and MSI-H was detected at this stage (Figure 3). Meanwhile, given the TMB-H and MSI-H status of the tumor, reimbursement for the immune checkpoint inhibitor pembrolizumab was approved by the health insurance, and pembrolizumab was started in February 2023.

**Figure 4.**
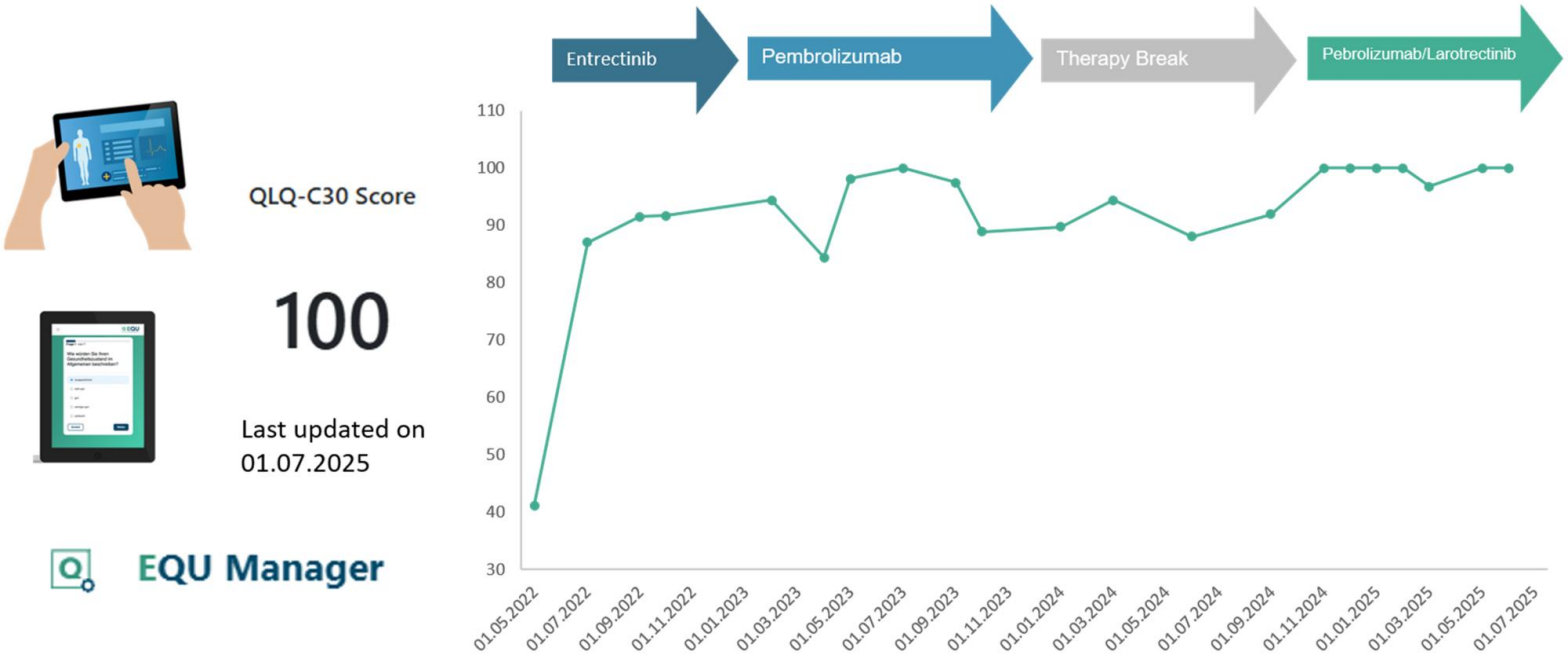
Durable clinical benefit reflected by longitudinal quality-of-life assessment. Quality of life (QoL) was monitored using the EORTC QLQ-C30 questionnaire as part of a prospective registry study that routinely includes patient-reported outcomes in individuals undergoing broad genomic profiling. Monthly assessments demonstrated substantial improvement following initiation of targeted therapy, with sustained high QoL scores throughout subsequent immunotherapy and combination treatment. The stability of patient-reported outcomes over more than 2 years reflects the durable clinical benefit achieved with personalized therapy in this rare molecular PDAC subtype.

Throughout the following months the patient experienced no treatment-related adverse events and remained in excellent clinical condition, enabling a full return to work. CT scans in May and September 2023 demonstrated partial response with reduced lymphadenopathy and residual peripancreatic tissue consistent with non-active fibrotic changes. This correlated with sustained molecular remission (Figure 3) and improved patient-reported outcomes (Figure 4).

In January 2024, a new pulmonary lesion was detected on CT. PET-CT in February 2024 suggested post-infectious changes, consistent with a recent respiratory infection. Due to the possibility of immune-related pneumonitis, corticosteroids were initiated, and pembrolizumab was stopped in March 2024. Subsequent febrile episodes and back pain led to the diagnosis of spondylodiscitis in May 2024, requiring antibiotic therapy for nearly three months. In September 2024, CT imaging showed unchanged residual pancreatic tissue and complete resolution of the pulmonary lesion.

Control LBs confirmed molecular remission until September 2024, when re-emergence of MSI-H and the *NTRK1* fusion indicated molecular relapse. At this time, the patient remained in clinical remission. To prevent further tumor growth, pembrolizumab was resumed in October 2024.

In January 2025, despite stable CT findings, LB showed a continued increase in allele frequencies under ongoing checkpoint inhibition (Figure 3). This resulted in the decision to resume NTRK inhibition as well in April 2025. In July 2025, the patient maintained a sustained clinical and molecular remission without evidence of tumor DNA on LB and with good quality of life under combined immune checkpoint and NTRK inhibition.

## Discussion

A hallmark of this case is ctDNA-based monitoring of two precision therapies to guide treatment in a poor-prognosis malignancy, supported by 13 serial liquid biopsies over 3 years and repeated MTB discussions.

The coexistence of MSI-H and an NTRK fusion in PDAC is rare and, to our knowledge, has not been reported previously. Microsatellite instability in pancreatic cancer is uncommon, with reported frequencies around 1%-2%.^4^ MSI-H status predicts responsiveness to immune checkpoint inhibition (ICI), now standard across multiple tumor types. NTRK fusions represent tissue-agnostic oncology with approved targeted inhibitors available. While NTRK1/2/3 fusions are most common in sarcomas, their prevalence in PDAC is below 1% but increases to 6% in *KRAS* WT tumors.^5^

Mismatch repair aberrations frequently co-occur with *NTRK* fusions, whereas oncogenic RAS or BRAF activation and *NTRK* fusions are typically mutually exclusive, as demonstrated in colorectal cancer cohorts.^6^ This suggests that NTRK-positive PDACs may represent a unique subtype with high TMB and increased likelihood of MSI-H.

The identification of TMB-H, MSI-H, and an NTRK fusion highlights the molecular landscape of this malignancy. Although each of these alterations is actionable, their coexistence raises the clinical question of appropriate treatment strategies and target prioritization. While experience from other entities often supports ICI initiation when both options are available,^7^ we chose the opposite sequence. Given the poor condition after chemotherapy failure, we prioritized NTRK inhibition, allowing rapid treatment and clinical stabilization. Immunotherapy was deferred due to anticipated delays in efficacy and reimbursement requirements. While ICIs often require longer to achieve clinical response, their effects tend to be more durable once established. This is evidenced by stabilized survival curves, higher long-term remission rates, and durable responses after treatment discontinuation.^8^^,^^9^

In the context of our case, this biological distinction between targeted therapy and ICI highlights the complementary potential of both strategies. NTRK inhibition provided rapid tumor control, which stabilized the patient clinically and may also have reduced tumor-mediated immunosuppression, creating a more favorable environment for subsequent immune checkpoint inhibition. Moreover, targeted therapy can disrupt oncogene “addiction,” potentially triggering tumor senescence and enhancing T-cell-mediated immune clearance, suggesting that a sequenced or combinatorial approach could maximize immediacy and durability of therapeutic responses.^10^

As molecular markers increasingly guide treatment strategies, the need for evaluation of treatment response has become important. MRD is an established indicator of tumor response and relapse in hematologic malignancies. In contrast, its role in solid tumors remains less defined.^3^ Rising levels of ctDNA represent a molecular hallmark of MRD and often precede radiological evidence of relapse. In PDAC, postoperative ctDNA detection provides strong evidence of residual disease and identifies patients with a high risk of recurrence.^11^ Our observations are consistent with data from previous trials demonstrating increased ctDNA and resistance development with a median lead time ranging from ∼20 days to 4 months before radiologic progression.^12–14^ Moreover, unlike most published reports that relied on a limited sample number (∼3-7),^15–17^ our case is characterized by 13 liquid biopsies, providing a detailed picture of tumor dynamics and heterogeneity.

From a biological point of view, this intensive monitoring allowed the detection of small molecular changes indicative of progression at an early time point, before radiological evidence of progression. Real-time monitoring of ctDNA during therapy can therefore identify emerging resistance or progression before it becomes evident through standard monitoring. A molecular relapse was detected during antibiotic treatment for spondylitis and a temporary ICI interruption. This might be linked to the fact that antibiotic use before or during ICI alters the microbiota repertoire and has an impact on disease course.^18^ In response to the continued increase in ctDNA, treatment was intensified by adding an NTRK inhibitor to the resumed immunotherapy. Preclinical data support this strategy, as there is evidence that NTRK inhibition can enhance the efficacy of anti-PD-1 therapy by increasing T-cell infiltration and altering the tumor microenvironment.^19^ Together, this highlights how MRD evaluation can complement follow-up and enable earlier therapeutic intervention. However, this personalized approach also has important limitations. High-frequency serial LB testing is associated with substantial cost and logistical effort, and reimbursement approval is not guaranteed. These factors may restrict the feasibility of such intensive monitoring in routine practice. In addition, ctDNA-based monitoring may be influenced by biological factors such as tumor burden and variable ctDNA shedding, which can affect detectability of molecular alterations. The very low concentrations of ctDNA in the MRD setting may require ultra-sensitive assays and can increase the risk of false-negative findings. Moreover, prospective interventional studies are still needed to determine how ctDNA-guided treatment decisions should be integrated into routine clinical practice.^3^

Nevertheless, the longitudinal liquid biopsy approach complemented the therapy sequencing strategy by providing real-time insights into tumor dynamics. It enabled MRD evaluation as well as the early detection of an acquired NTRK resistance mutation. This is relevant in NTRK-driven PDAC, where fusions with varying partners have been documented, and NTRK inhibition resulted in positive outcomes.^20^^,^^21^ Notably, NTRK-positive patients usually relapse within the first year of treatment, indicating that resistant clones become dominant quickly.^22^

Somatic mutations in the kinase domain of *NTRK1/2/3*, conferring resistance to the type I inhibitors larotrectinib and entrectinib, have been identified in a number of NTRK fusion-positive tumors.^23^ Next-generation inhibitors (eg, selitrectinib, repotrectinib) maintain activity against secondary resistance tumors.^24^^,^^25^ However, given the limited availability of tumor tissue and the high tumor heterogeneity, repeated biopsies are often not feasible for capturing clonal evolution. In this context, it is extremely important to find a rapid and reliable technique to detect resistance and define new therapeutic vulnerabilities, such as serial ctDNA-based monitoring.

## Author contributions

Sarah Rohlfing (Conceptualization [true], Investigation [true], Writing Original Draft [true], Writing Review Editing [true]), Dilyana Vladimirova (Project Administration [true], Visualization [true], Writing Original Draft [true], Writing Review Editing [true]), Stephanie Berger (Conceptualization [true], Visualization [true], Writing Review Editing [true]), Sylvia Bochum (Data Curation [true], Formal Analysis [true], Writing Review Editing [true]), Gabrijela Otmacic (Data Curation [true], Writing Review Editing [true]), Marlene Weiß (Investigation [true], Writing Review Editing [true]), Bence Sipos (Data Curation [true], Formal Analysis [true], Writing Review Editing [true]), Saskia Biskup (Data Curation [true], Formal Analysis [true], Writing Review Editing [true]), Marion Klaumünzer (Data Curation [true], Formal Analysis [true], Writing Review Editing [true]), and Uwe M. Martens (Funding Acquisition [true], Investigation [true], Supervision [true], Writing Review Editing [true])

## Funding

The case report was funded by a research grant from the Marianne & Teodor Dauenhauer Stiftung.

## Conflicts of interest

All authors have completed and signed the conflict of interest disclosure form. The authors declare that the following competing interests exist: U.M.M. is a founder and CEO of the MOLIT Institute gGmbH which is supported by the Dieter Schwarz Foundation. He is Chairman of the Cancer Society Baden-Württemberg. U.M.M. reports consulting fees by BMS, Sanofi, Pierre Fabre, Astellas, Takeda, MSD, IOMEDICO and Roche; honoraria for lectures/presentations by BMS, Guardant Health; support for attending meetings and/or travel by Lilly, Amgen, Roche, BMS, BeOne, Ipsen, Daiichi-Sankyo and Pierre Fabre. S.R. has received travel expense support from Merck and Grifols. All other authors declare that they have no competing interests.

## Data Availability

All data are included in this article. Additional information is available from the corresponding author upon reasonable request.
